# ERβ alters the chemosensitivity of luminal breast cancer cells by regulating p53 function

**DOI:** 10.18632/oncotarget.25147

**Published:** 2018-04-27

**Authors:** Igor Bado, Eric Pham, Benjamin Soibam, Fotis Nikolos, Jan-Åke Gustafsson, Christoforos Thomas

**Affiliations:** ^1^ Department of Biology and Biochemistry, Center for Nuclear Receptors and Cell Signaling, University of Houston, Houston, Texas, USA; ^2^ Department of Biology and Biochemistry, University of Houston, Houston, Texas, USA; ^3^ Department of Computer Science and Engineering Technology, University of Houston-Downtown, Huston, Texas, USA; ^4^ Center for Innovative Medicine, Department of Biosciences and Nutrition, Karolinska Institutet, Huddinge, Sweden

**Keywords:** estrogen receptor β, p53, breast cancer, DNA damage response, therapy response

## Abstract

Estrogen receptor α (ERα)-positive breast cancers tend to develop resistance to both endocrine therapy and chemotherapy. Despite recent progress in defining molecular pathways that confer endocrine resistance, the mechanisms that regulate chemotherapy response in luminal tumors remain largely elusive. Luminal tumors often express wild-type p53 that is a major determinant of the cellular DNA damage response. Similar to p53, the second ER subtype, ERβ, has been reported to inhibit breast tumorigenesis by acting alone or in collaboration with p53. However, a synergistic mechanism of action has not been described. Here, we suggest that ERβ relies on p53 to elicit its tumor repressive actions in ERα-positive breast cancer cells. Upregulation of ERβ and treatment with ERβ agonists potentiates the tumor suppressor function of p53 resulting in decreased survival. This effect requires molecular interaction between the two proteins that disrupts the inhibitory action of ERα on p53 leading to increased transcriptional activity of p53. In addition, we show that the same interaction alters the chemosensitivity of endocrine-resistant cells including their response to tamoxifen therapy. Our results suggest a collaboration of ERβ and p53 tumor suppressor activity in breast cancer cells that indicates the importance of ligand-regulated ERβ as a tool to target p53 activity and improve the clinical management of resistant disease.

## INTRODUCTION

Nearly 70% of diagnosed breast cancers belong to estrogen receptor alpha (ERα)-positive phenotype [1, 2]. Treatment with the antiestrogen tamoxifen that alters the conformation of ERα that is induced by 17β-estradiol is the standard treatment option for these tumors [3]. However, *de novo* and acquired resistance to endocrine therapy is developed in 50% of the cases [4]. Only part of the mechanism that links estrogen signaling to therapy resistance has been elucidated including the altered expression and/or post-translational modification of ERα that results in aberrant activity [5]. The discovery of ERβ indicated the complexity of estrogen signaling and suggested the possibility of the second ER to interfere with the pathways that contribute to resistant phenotypes. Both ERα and ERβ are transcription factors that regulate a plethora of genes by acting on estrogen-response-elements (ERE) or by interacting with other transcription factors [5, 6]. Despite similarities in the structure and the mechanism of action, the two ER subtypes elicit distinct transcriptional responses and differentially affect cancer cellular processes which may imply separate roles in therapy resistance.

In addition to estrogen receptor activity, other factors that regulate cell survival have been associated with therapy resistance in breast cancer. Among these, the p53 protein that is expressed in its wild-type form in approximately 80% of ERα-positive breast cancers [8, 9]. As a tumor suppressor, p53 regulates cell-cycle arrest, DNA repair, apoptosis and senescence through induction of downstream effectors including cyclin-dependent kinase inhibitor 1 (p21^WAF1^), growth arrest and DNA-damage-inducible alpha (GADD45A), p53 upregulated modulator of apoptosis (PUMA), BCL-2-like protein 4 (BAX), plasminogen activator inhibitor-1 (PAI-1), and NOXA [10–13]. In response to stress, p21 promotes G_1_/S cell cycle arrest [14] and the BCL-2 family member PUMA induces apoptosis by primarily activating the pro-apoptotic proteins BAX and/or BAK in mitochondria [15]. Upon genotoxic stress, GADD45A induces growth arrest and apoptosis by interacting with p21 and CDC2 and PAI-1 is essential for replicative senescence [16–20]. In addition to downstream effectors, regulators of p53 expression and activity affect its tumor suppressor function. In response to DNA damage, ATM and ATR upregulate p53 through phosphorylation that disturbs its interaction with the ubiquitin ligase MDM2. Upregulation of MDM2 in breast carcinomas results in accelerated p53 degradation and is associated with worse prognosis [21–24]. Similar to MDM2, the ubiquitin ligase MDMX directly impedes p53 transcriptional activity or heterodimerizes with MDM2 to induce p53 degradation [25]. Consequently, due to its pivotal impact on cell survival signaling, deregulation of the p53 pathway is an important step in the process that leads to resistant tumor phenotypes [26, 27]. Altered activity of this pathway has been associated with resistance to ER-targeted therapies and chemotherapies [28]. However, what signaling mitigates wild-type p53 activity in ERα-positive tumors is still poorly understood.

Activation of the p53 pathway has been inversely associated with ERα activity in breast cancer. While ERα levels increase during the development of breast cancer, p53 expression is lower in luminal tumors compared with the normal mammary gland [29]. The inverse association between the two proteins reflects their opposite roles during malignant transformation and may account for the early onset breast tumors that are induced by exogenous estrogen in absence of p53 [30]. At the molecular level, despite the proposed involvement of ERα in regulation of p53 expression [31], the receptor is likely to act on p53 transcriptional activity. ERα was indeed found to bind to and repress p53-depedent transcription and its associated tumor suppressor function [32–34] and disruption of this interaction by radiation restores p53 function [35, 36]. In contrast to ERα and similar to p53 downregulation, ERβ expression decreases in breast cancer [37, 38]. The reduced levels of the two proteins in human tumors may explain the observed collaboration of ERβ and p53 inactivation in mouse breast tumor development [37]. This may imply an ERβ-p53 transcriptional cooperation that inhibits tumor-associated phenotypes. ERβ has so far been shown to interact with and inhibit the pro-invasive properties of mutant p53 [7]. Thus, the p53 tumor suppressor activity in breast cancer may be differentially regulated by the two ER subtypes when both are expressed in cancer cells [39, 40]. In such cellular context, by heterodimerizing with ERα, ERβ can oppose the pro-survival function of ERα [41–44]. Despite that aspects of the molecular estrogen receptor-p53 associations are not completely understood, it is evident that the p53 pathway is regulated by estrogen and adjusting ER activity with ER-subtype specific ligands may control p53-dependent tumor suppressor function. The objective of the present study was to investigate whether ERβ transcriptionally cooperates with p53 to impact survival and chemosensitivity of luminal breast cancer cells. Here, we show that ERβ enhances wild-type p53 transcriptional activity proposing a new mechanism that is employed by the receptor to elicit tumor repressive actions in breast cancer.

## RESULTS

### ERβ regulates p53 transcriptional activity

**Figure 1:**
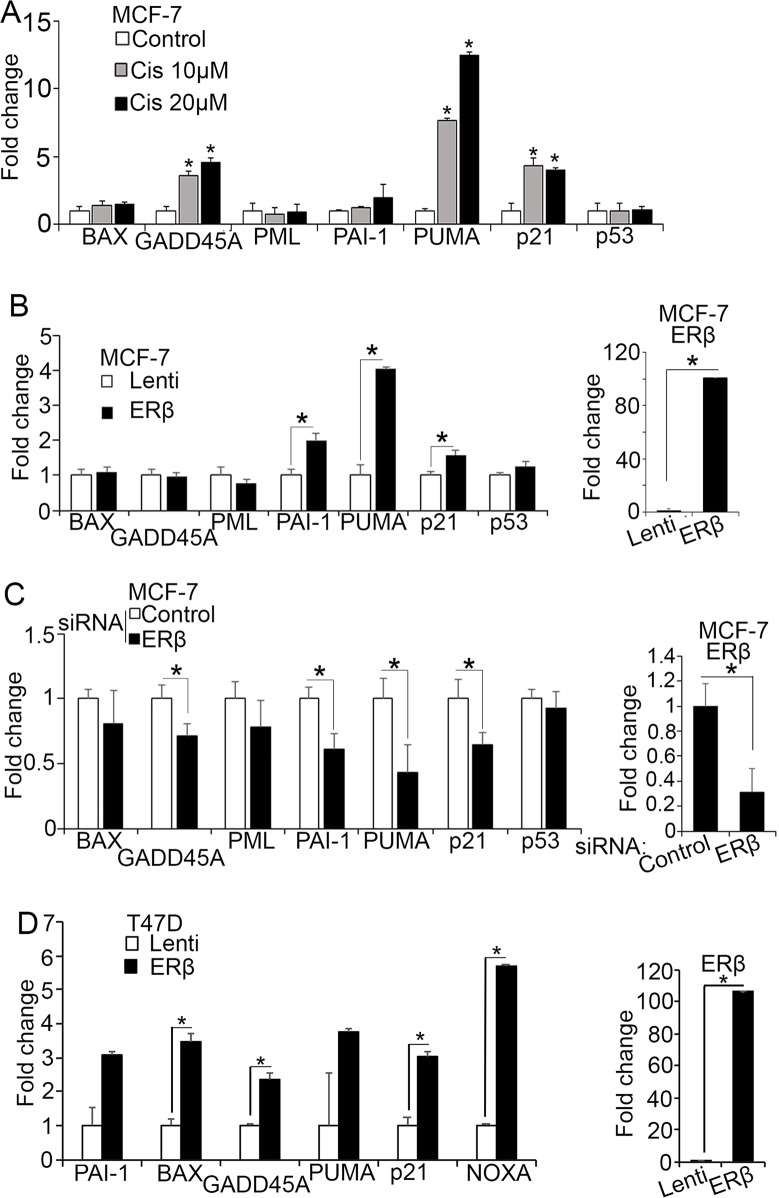
ERβ increases p53 transcriptional activity **(A)** MCF-7 cells were left untreated (Control) or treated with 10 μM or 20 μM cisplatin for 24 hours and mRNA expression of p53 target genes was analyzed by real-time PCR. Values were normalized to that of the untreated cells that was set to 1. **(B)** mRNA expression of p53 target genes in control (Lenti) and ERβ-expressing MCF-7 cells. Values were normalized to control cells. **(C)** mRNA expression of p53 target genes in MCF-7 cells after transfection with control or siRNA#1 against ERβ. **(D)** mRNA levels of p53 target genes in control (Lenti) and ERβ-expressing T47D cells. In all graphs, values represent the mean ± S.D. of three independent experiments; ^*^P ≤ 0.05.

The expression of full length ERβ has been associated with better survival in breast cancer [45–47]. Despite the proposed mechanisms of action, it is still poorly understood how the receptor is linked to less aggressive tumor phenotypes [6, 36, 47, 44–46]. ERβ deletion has recently been reported to collaborate with p53 inactivation to induce early onset breast tumors in mice [37] suggesting that ERβ synergizes with wild-type p53 to elicit anti-tumor activities in breast cancer cells. To test whether such synergism impacts the clinical outcome of patients with breast cancer, we tested the correlation between the combined expression of ERβ and p53 and relapse free survival in published Kaplan Meier (KM) plotter datasets and found that ERβ^high^/p53^high^ patients have better prognosis than ERβ^low^/p53^low^ patients in ERα-positive breast cancer cohort (Supplementary Figure 1A). To investigate whether ERβ relies on such synergism to exert its repressive actions in breast cancer, we analyzed breast cancer cells that carry wild-type p53 for expression of p53-regulated genes that are involved in cell-cycle arrest, apoptosis, and senescence including p21, GADD45A, PUMA, PAI-1, BAX, and promyelocytic leukemia protein (PML). To ascertain the functionality of p53 in ERα-positive MCF-7 cells, we measured the expression of these genes after exposing the cells to genotoxic stress imposed by the DNA-crosslinking agent cisplatin. By inducing DNA-damage, cisplatin stabilizes p53 promoting its nuclear translocation and transcriptional activation [49]. Consistent with previous studies, a strong upregulation of PUMA, GADD45A, and p21 mRNA was observed in MCF-7 cells after treatment with 10 μM cisplatin confirming the link between DNA damage and p53 activation [11]. PAI-1 was slightly induced only after treatment with a higher drug concentration (20 μM), while BAX that is post-transcriptionally regulated by cytoplasmic p53 through a mitochondria-dependent mechanism and PML did not respond to treatment (Figure 1A) [50]. Similar to cisplatin, upregulation of ERβ in MCF-7 cells significantly induced the expression of PUMA, PAI-1 and p21 but not GADD45A (Figure 1B). To corroborate the effects of ERβ on p53 transcriptional activity, we analyzed the mRNA levels of the same p53 target genes after transiently transfecting MCF-7 cells with siRNA [48] that silences ERβ. Downregulation of ERβ decreased the expression of all genes that were upregulated in ERβ-transfected MCF-7 cells (PAI-1, PUMA, p21) including GADD45A (Figure 1C). Analysis of publically available chromatin immunoprecipitation (ChIP) sequencing data revealed a strong co-enrichment of ERβ and p53 at regulatory elements of several p53 target genes including GADD45A in MCF-7 cells (Supplementary Figure 1B and Supplementary Table 1). Due to strong promoter binding, the transcriptional activity of endogenous ERβ in MCF-7 cells can account for increased levels of GADD45A that are not further affected by the transfected receptor explaining the alteration of GADD45A mRNA only upon ERβ knockdown. ERβ was previously shown to interact with mutant p53 in triple-negative breast cancer (TNBC) cells altering the expression of mutant p53-associated genes that regulate invasion [7]. To test whether a similar interaction in luminal cells affects the expression of p53 target genes that influence survival and apoptosis, we analyzed ERα-positive T47D cells that express mutant p53. As shown in Figure 1D, upregulation of ERβ in these cells significantly increased the expression of the p53 target genes BAX, GADD45, PUMA and NOXA suggesting that ERβ can activate the wild-type function of mutant p53. Re-activation of mutant p53 was previously demonstrated by small molecules that affect its interaction with other proteins [51]. Taken together, these results strengthen our hypothesis that ERβ plays a crucial role in regulating p53 transcriptional activity.

### ERβ enhances p53 activity in response to genotoxic stress

**Figure 2:**
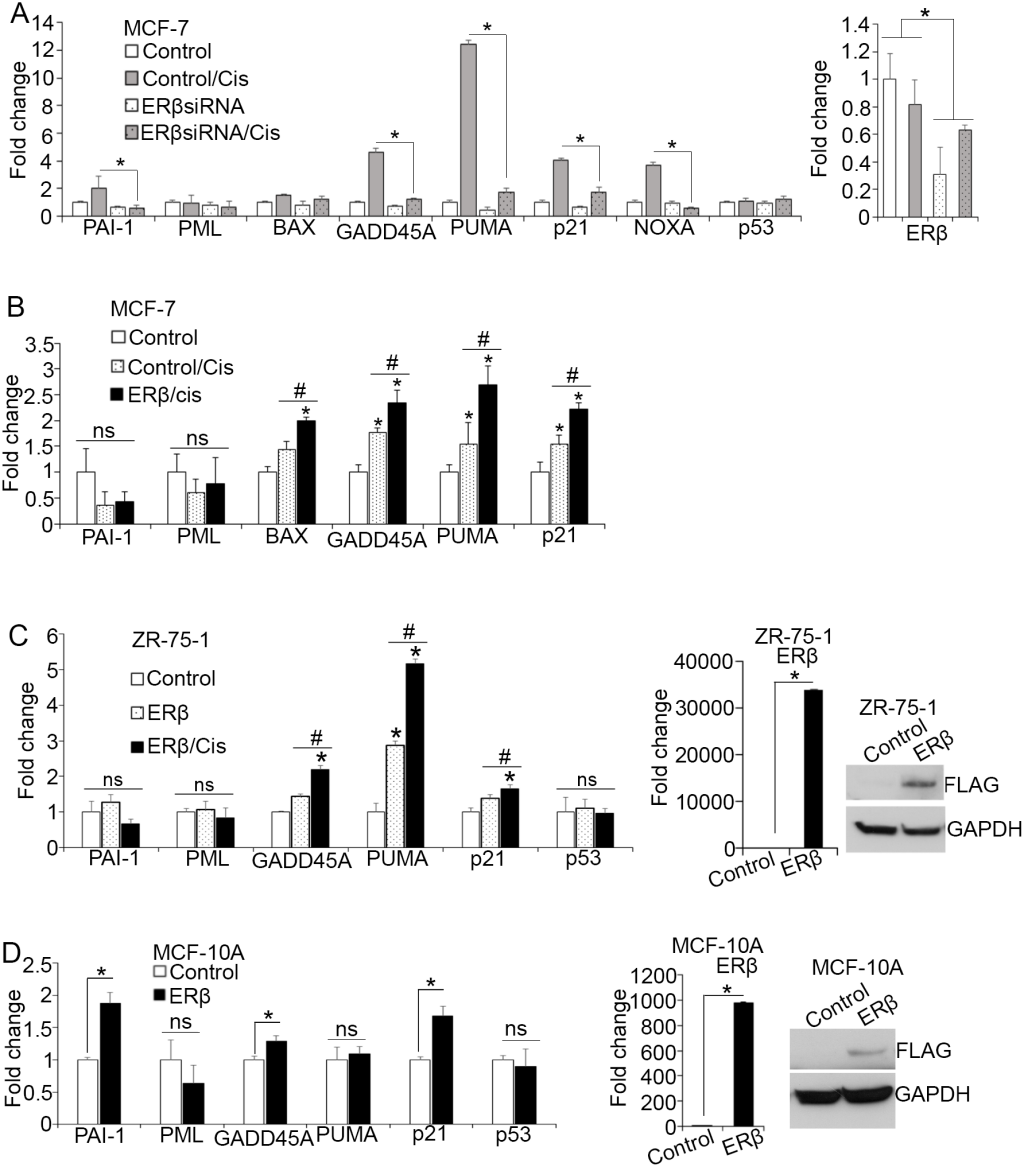
ERβ alters p53-dependent transcription in response to chemotherapy **(A)** mRNA levels of p53 target genes in MCF-7 cells following transfection with control (Control) or siRNA#1 against ERβ and treatment with vehicle or 20 μM cisplatin for 24 hours. Values were normalized to the untreated control cells. **(B)** mRNA expression of p53 target genes in control and ERβ-expressing MCF-7 cells following treatment with vehicle or 20 μM cisplatin. Values were normalized to the untreated control cells. **(C)** Left: mRNA levels of p53 target genes in control and ERβ-expressing ZR-75-1 cells after treatment with vehicle or 20 μM cisplatin. Right: mRNA and protein levels of ERβ in control and ERβ-expressing ZR-75-1 cells. **(D)** Left: Expression of p53 target genes in control and ERβ-expressing MCF-10A cells. Right: mRNA and protein levels of ERβ in control and ERβ-expressing MCF-10A cells. In all graphs, values represent the mean ± S.D. of three experiments; ^*^P ≤ 0.05.

By inducing DNA damage response, cisplatin stabilizes p53 protein promoting its activity [52]. To examine whether ERβ affects chemotherapy-induced p53 tumor suppressor function, MCF-7 cells were analyzed for p53-dependent gene expression following ERβ downregulation using two different siRNAs and treatment with cisplatin for 24 hours. As shown in Figure 2A and Supplementary Figure 2, downregulation of ERβ prevented the cisplatin-induced expression of these genes, indicating that ERβ contributes to activation of wild-type p53 in response to genotoxic stress in luminal breast cancer cells. To validate our findings, we measured p53-dependent gene expression following upregulation of ERβ in the presence of chemotherapy. Our results revealed significant increase in the expression of p53 target genes after upregulation of ERβ in cisplatin-treated MCF-7 cells (Figure 2B). Importantly, the expression of BAX that was not affected by either cisplatin alone or ERβ upregulation (Figure 1A and 1B), increased following combined treatment, suggesting a synergistic ERβ-p53 function (Figure 2B). To corroborate our findings, we evaluated the effects of ERβ on ZR-75-1 cells that represent another cell model of ERα-positive breast cancer that expresses wild-type p53 protein. Consistent with the MCF-7 cells, induction of ERβ expression in ZR-75-1 cells significantly upregulated most of the p53 target genes in absence and presence of chemotherapy demonstrating the importance of ERβ in enhancing p53 transcriptional activity under basal conditions or in response to genotoxic stress (Figure 2C). To investigate the clinical importance of these common ERβ and p53 target genes, we examined whether their expression is associated with relapse-free survival in published KM plotter datasets [53]. As shown in Supplementary Figure 1C, increased expression of GADD45A, PUMA, p21 and PAI-1 correlates with better prognosis in patients with ERα-positive breast cancer after endocrine therapy and chemotherapy. Given, that both MCF-7 and ZR-75-1 cells express significant amount of ERα, the formation of ERα-ERβ heterodimers may account for the ability of ERβ to regulate the function of p53. Hence, we sought to determine whether ERβ can affect p53 transcriptional activity in ERα-deficient and non-tumorigenic mammary epithelial MCF-10A cells. Upregulation of ERβ in these cells increased the expression of the p53 target genes GADD45, p21 and PAI-1 but not PUMA that was strongly upregulated in ERα-positive breast cancer cell lines (Figure 2D). Given the specific pro-apoptotic function of PUMA, these results indicate a selective pro-apoptotic effect of the receptor in breast cancer cells. In addition, the ERβ-mediated increased expression of p53-target genes in MCF-10A cells suggests that ERβ can influence p53 function independently of ERα.

### ER ligands modulate p53 function

**Figure 3:**
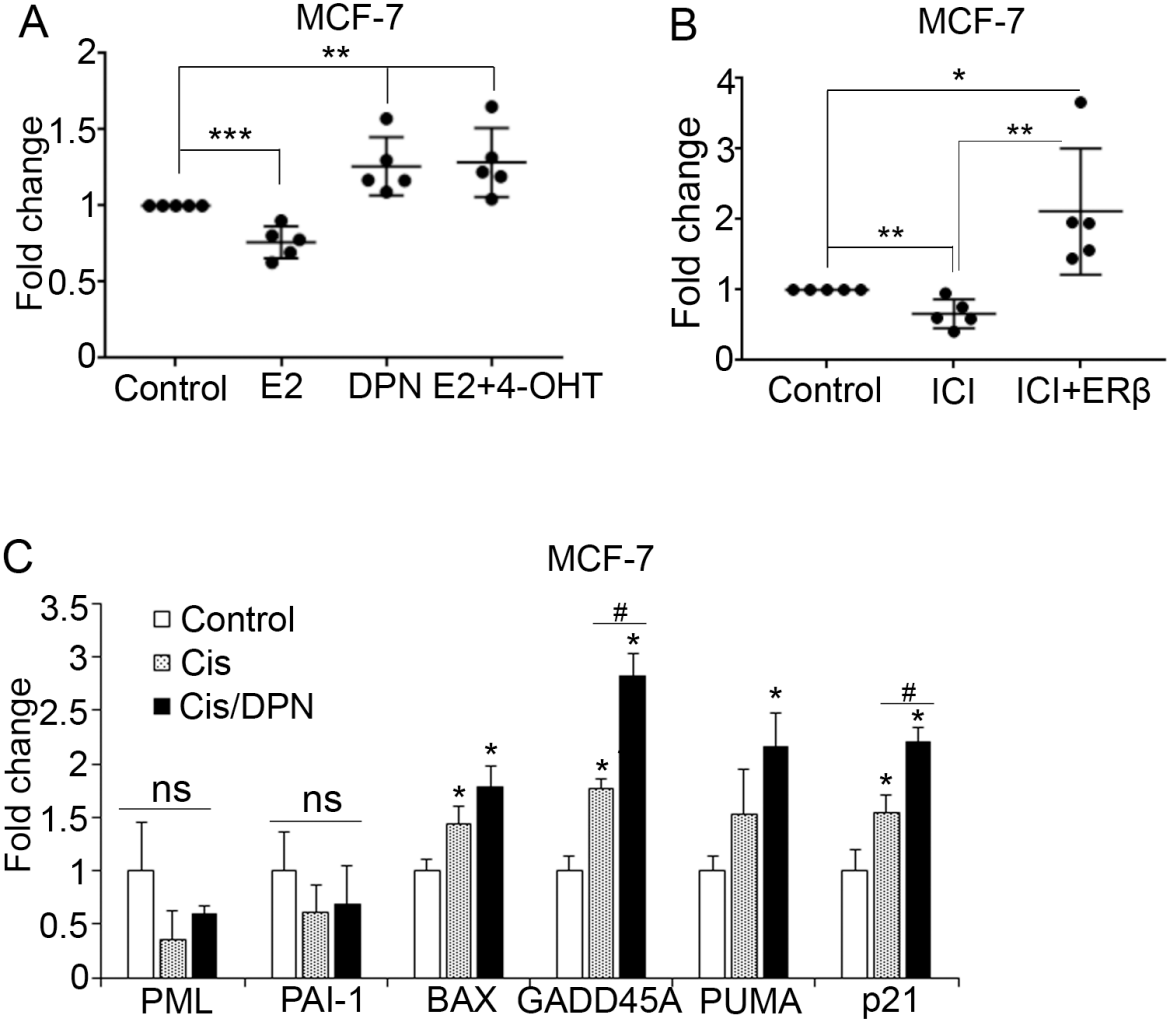
ER ligands modulate p53 function **(A)** Dot blot depicting the fold change of mRNA expression of p53 target genes in MCF-7 cells following treatment with vehicle (Control) or the ER subtype-specific ligands 17β-estradiol (E2, 10 nM), Diarylpropionitrile (DPN, 10 nM), or combination of E2 and 1 μM 4-hydroxytamoxifen (4-OHT) for 24 hours. Values were normalized to the untreated cells. **(B)** Dot blot representing the fold change of mRNA expression of p53 target genes in control and ERβ-expressing MCF-7 cells after treatment with 10 nM fulvestrant (ICI) for 24 hours. **(C)** mRNA levels of p53 target genes in MCF-7 cells following treatment with vehicle (Control), 20 μM cisplatin (Cis) or combination of 20 μM cisplatin and 10 nM DPN. Values were normalized to the untreated cells. In all graphs values represent the mean ± S.D. of three different experiments; ^*^P ≤ 0.05.

To ascertain the effect of endogenous ERβ on inducing p53 tumor suppressive activity, we evaluated the impact of ER subtype-specific ligands on the expression of p53-regulated genes in ERα-positive MCF-7 cells. Among these compounds, 17β-estradiol (E2) binds to and activates both receptors and its growth stimulatory effects are linked to activation of the pro-survival ERα that is expressed in higher levels in luminal cancer cells compared with ERβ [7]. Consistent with its pro-survival action, E2 reduced the expression of p53 target genes, apparently through the activation of ERα that is known to inhibit p53 transcriptional activity (Figure 3A) [27, 35]. Treatment of the cells with the ERα-specific antagonist 4-hydroxytamoxifen (4-OHT) reversed the E2-reduced expression of the same genes (Figure 3A), indicating the importance of ERα in mediating the effect of E2 on p53 transcriptional activity in ERα-positive breast cancer cells. To study the effects of specific activation of ERβ, cells were exposed to diarylpropionitrile (DPN), a selective ERβ agonist. As expected, DPN enhanced the expression of p53 target genes in MCF-7 cells (Figure 3A). In addition to tamoxifen, the selective ERα degrader fulvestrant (ICI182780) inhibits the growth stimulatory actions of estrogen [54]. In the absence of E2, treatment of MCF-7 cells with ICI182780 caused a decrease in the expression of p53 target genes (Figure 3B). In addition to acting as an ERα antagonist, ICI182780 has been shown to induce ERβ-mediated tumor repressive actions [55, 56]. Based on this evidence, we examined whether upregulation of ERβ alters the effect of ICI182780 on p53-depedent gene expression. Indeed, induction of ERβ expression in ICI-treated MCF-7 cells significantly upregulated the p53-regulated genes (Figure 3B), suggesting that ICI182780 can act as an ERβ agonist on p53-dependent gene expression in luminal cells. Moreover, as ERβ correlates with better response to chemotherapy in breast cancer [45], we investigated whether the ERβ-specific agonist DPN enhances p53 tumor suppressor activity in chemotherapy-treated cells. Treatment with DPN significantly potentiated the effect of cisplatin on p53-dependent gene transcription (Figure 3C), indicating a synergism between ERβ and p53 that may account for some of their previously observed anti-tumor effects in breast cancer [31, 37].

### ERβ increases the chemotherapy sensitivity of ERα-positive breast cancer cells

**Figure 4:**
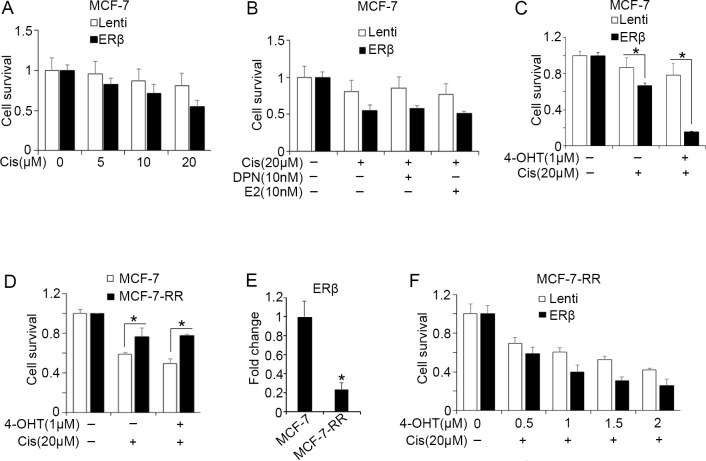
ERβ alters the chemosensitivity of breast cancer cells Survival of breast cancer cells was analyzed using MTS assay following treatment with the indicated drugs for 72 hours. **(A)** Survival of control (Lenti) and ERβ-expressing MCF-7 cells after treatment with the indicated concentrations of cisplatin. **(B)** Survival of control (Lenti) and ERβ-expressing MCF-7 cells after treatment with 10 nM DPN or 10 nM estradiol (E2) in the presence of 20 μM cisplatin (Cis). **(C)** Survival of control (Lenti) and ERβ-expressing MCF-7 cells after treatment with 1 μM 4-OHT alone or in combination with 20 μM cisplatin. **(D)** Survival of tamoxifen-sensitive (MCF-7) and -resistant (MCF-7-RR) breast cancer cells following treatment with 1 μM 4-OHT with or without 20 μM cisplatin. **(E)** mRNA levels of ERβ in MCF-7 and MCF-7-RR cells. Values were normalized to MCF-7 cells. **(F)** Survival of control (Lenti) and ERβ-expressing MCF-7-RR cells following treatment with increasing concentrations of 4-OHT in the absence and presence of 20 μM cisplatin. Values represent the mean ± S.D. of three different experiments.

Based on our findings showing that ERβ increases the transcriptional activity of p53 in breast cancer cells, we investigated whether the receptor alters the chemosensitivity of these cells. We first determined the sensitivity of chemotherapy-treated MCF-7 cells following upregulation of ERβ. As shown in Figure 4A, cisplatin treatment caused a significantly stronger decrease in the survival of ERβ-expressing compared with the control MCF-7 cells. This effect may reflect the increased expression of p53 target genes that was observed following upregulation or activation of ERβ in cisplatin-treated cells (Figures 2 and 3C). The survival of the cisplatin-treated, ERβ-expressing MCF-7 cells was not further altered by E2 or the ERβ-specific agonist DPN (Figure 4B). In contrast, a dramatic increase in the sensitivity of the same cells was observed after treatment with tamoxifen suggesting an association of ERβ with response to endocrine therapy following p53 upregulation (Figure 4C). To investigate whether a similar association occurs in the clinical setting, we tested the correlation of ERβ and p53 co-expression with relapse-free survival in published KM plotter datasets [53]. As shown in Supplementary Figure 1D, ERβ^high^/p53^high^ breast cancer patients have better clinical outcome than ERβ^low^/p53^low^ patients following therapy with tamoxifen. In addition, we stratified ERα-positive/HER2-negative patients that respond better to endocrine therapy in those with tumors that have wild-type p53 and any p53 status. High ERβ expression was associated with significantly better survival only in patients with wild-type p53 indicating that the presence of a functional p53 is important for the anti-tumor activity of ERβ (Supplementary Figure 1E and 1F).

Given that ERα-positive tumors tend to develop resistance to tamoxifen treatment and ERβ associates with tamoxifen sensitivity of chemotherapy-treated ERα-positive cells, we examined whether ERβ alters responses of tamoxifen-resistant MCF-7-RR cells to chemotherapy and/or endocrine therapy. We initially observed increased resistance of MCF-7-RR cells to cisplatin treatment compared with wild-type MCF-7 cells, suggesting development of cross-resistance to both endocrine therapy and chemotherapy (Figure 4D). In addition, cisplatin treatment did not restore sensitivity of MCF-7-RR cells to 4-hydroxytamoxifen (Figure 4D) [57]. Given that MCF-7-RR cells express substantially less ERβ than the tamoxifen-sensitive MCF-7 cells [58] (Figure 4E), we examined whether upregulation of the receptor in the presence of cisplatin restores their sensitivity to tamoxifen. As shown in Figure 4F, induction of ERβ expression increased the sensitivity of cisplatin-treated MCF-7-RR cells to 4-hydroxytamoxifen.

### ERβ interacts with wild-type p53

**Figure 5:**
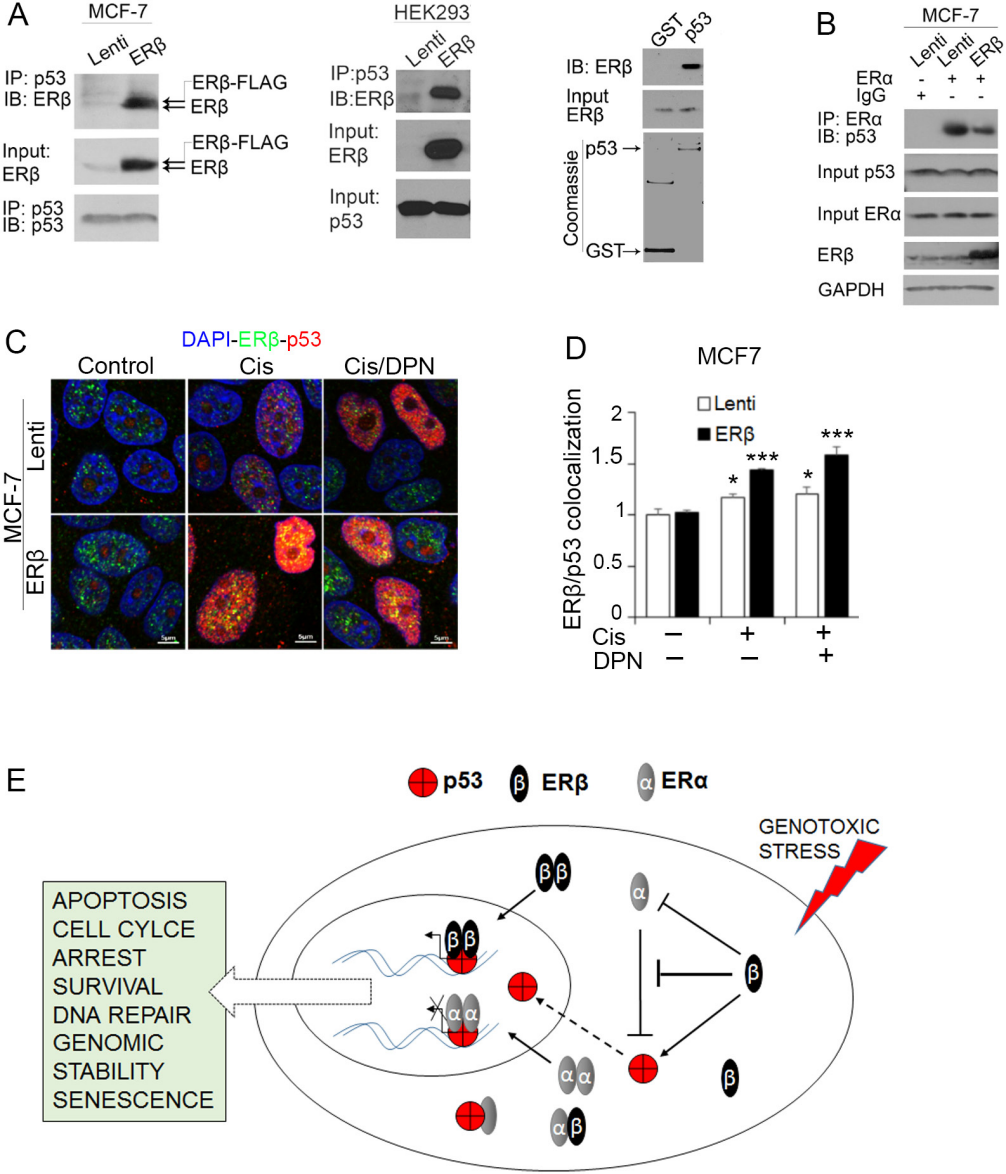
ERβ interacts with wild-type p53 **(A)** Left: Co-immunoprecipitation of p53 showing ERβ interaction with p53 in control (Lenti) and ERβ-expressing (ERβ) MCF-7 cells. Middle: Co-immunoprecipitation of p53 showing ERβ interaction with p53 in control (Lenti) and ERβ-expressing (ERβ) HEK-293T cells. Right: Bacteria-produced GST-tagged p53 (WT p53) proteins were used to pull-down flag-tagged *in vitro* translated ERβ and all samples were denatured and used for electrophoresis. **(B)** Co-immunoprecipitation of ERα showing ERα interaction with p53 in control (Lenti) and ERβ-expressing MCF-7 cells. **(C)** Immunofluorescence imaging of ERβ (green) and p53 (red) in control (Lenti) and ERβ-expressing MCF-7 cells following treatment with 20 μM cisplatin alone (Cis) or together with DPN (Cis/DPN) for 24 hours. Nuclei were stained with DAPI (Blue). **(D)** Quantification of ERβ and p53 co-localization in control (Lenti) or ERβ-expressing MCF-7 cells. **(E)** Scheme representing the synergistic p53 and ERβ tumor suppressor function in ERα-positive breast cancer cells.

To better understand the molecular mechanism that is employed by ERβ to regulate wild-type p53 function, we examined whether the two proteins interact. We previously showed that ERβ binds to the intact C-terminus of p53 proteins carrying missense mutations in their DNA-binding domain [7]. The interacting domain of mutant p53 gave us a hint of a potential binding of ERβ to wild-type p53. To examine whether an association of ERβ with wild-type p53 occurs in breast cancer cells, we carried out co-immunoprecipitation (CoIP) experiments in wild-type p53-expressing MCF-7 cells after upregulation of ERβ. In MCF-7 cells that express low levels of endogenous ERβ, both the transfected and endogenous receptors were found to interact with p53 (Figure 5A, left). This interaction was also observed in HEK-293 cells (Figure 5A, middle). In addition to CoIP, GST-pull down assay revealed a direct p53-ERβ binding further supporting the interaction between the two proteins (Figure 5A, right).

Given that MCF-7 cells express high levels of ERα that binds to and inhibits the activity of p53 [9], we examined whether ERβ enhances p53 activity in these cells by affecting the interaction of ERα with p53. CoIP experiments showed a substantially lower ERα-p53 association in ERβ-expressing compared with the control cells (Figure 5B). These results suggest that ERβ may enhance the tumor suppressor function of p53 in luminal breast cancer cells by preventing the inhibitory effect of ERα on p53.

Nuclear accumulation of p53 is essential for its transcriptional activation in response to DNA damage [59]. To examine whether ERβ alters p53 subcellular localization, control and ERβ-expressing MCF-7 cells were exposed to cisplatin alone or cisplatin together with the ERβ agonist DPN and analyzed by confocal microscopy. Upregulation of ERβ and/or treatment with DPN enhanced the cisplatin-induced accumulation of p53 in the nucleus of the cells. A co-localization of p53 and ERβ was also detected under the same conditions (Figure 5C and 5D) that is consistent with the p53 transcriptional activation following the interaction between the two proteins (Figures 1, 2 and 3C). In addition to changes in subcellular localization, induction of ERβ expression or treatment with DPN caused a remarkable increase in the levels of nuclear p53 (Figure 5C). Taken together, these results suggest that, in addition to acting as co-activator, ERβ may increase p53 activity by promoting its stability and nuclear translocation. They also indicate the potential of ERβ ligands to increase chemotherapy sensitivity of luminal breast cancer cells that express wild-type p53.

## DISCUSSION

Collaboration between ERβ and p53 inactivation has been shown to induce breast tumorigenesis [37]. This led us to hypothesize that ERβ may synergize with p53 to inhibit breast cancer cell growth and alter response to therapy. Our study identifies ERβ as a novel activator of wild-type p53-dependent transcription and this function results in decreased survival of luminal breast cancer cells. This mechanism of action may account for the observed association of ERβ with better prognosis in patients with breast cancer [60, 61]. In addition, we show that, by potentiating the chemotherapy-induced tumor suppressor activity of p53, upregulation of ERβ or activation with agonists increases the chemosensitivity of luminal breast cancer cells as it was previously shown with TNBC and lung cancer cells [62, 63]. Given that p53 is a master regulator of DNA damage response, by altering the activity of p53, ERβ seems to regulate signaling that determines the response of cells to chemotherapy-induced DNA damage. This is consistent with previously published data indicating involvement of the receptor in the regulation of DNA damage response pathways [62, 63]. The effect of ERβ under genotoxic stress may also explain recent published data that correlate the expression of the receptor in breast tumors with better response to chemotherapy [45]. These associations indicate a potential predictive role of ERβ in defining patients with functional p53 protein that may benefit from chemotherapy.

Hormonal therapy is the primary option for treating ERα-positive breast cancers. However, a significant proportion of these tumors become resistant to endocrine compounds [41]. Pathways that are overexpressed in anti-estrogen resistant cells are also associated with chemotherapy resistance [58, 64, 65]. In support of these findings, our results suggest that tamoxifen-resistant cells are more resistant to cisplatin, suggesting the development of a cross-resistant cancer cell phenotype. Ligand-independent activation of ERα and aberrant activity of molecular signaling pathways that regulate survival and apoptosis including the p53 pathway are implicated in endocrine resistance of ERα-positive breast tumors [64]. Despite that luminal tumors often maintain wild-type p53 alleles, perturbation of the p53 tumor suppressive function is associated with more aggressive disease status [66]. One of the mechanisms that account for the deregulation of the p53 pathway in these tumors relies on its interaction with ERα. This interaction was shown to inhibit p53-dependent apoptosis in breast cancer cells by impeding the nuclear translocation and transcriptional activity of p53 [36]. The therapeutic potential of this association was demonstrated when ionizing radiation was found to disrupt the ERα-p53 interaction allowing p53 to resume its function [36]. In contrast to ERα, upregulation of ERβ has been shown to affect the survival of breast cancer cells in a similar manner as wild-type p53 including effects on cell cycle regulators, growth factor receptor and stress response signaling pathways [6, 47, 56]. ERβ has also been proposed to act on endocrine-resistant phenotypes. Despite the association of the receptor with decreased survival of tamoxifen-resistant cells [58], its role in endocrine resistance is still not well defined. We observed here that in agreement with the decreased expression of ERβ in tamoxifen-resistant compared with the -sensitive luminal cells, upregulation of ERβ in endocrine-resistant cells decreases the survival in response to chemotherapy or combined chemotherapy and endocrine therapy. We also investigated the mechanism that is employed by the receptor to elicit these tumor repressive actions. Previous studies have shown that ERβ forms heterodimers with ERα that result in inhibition of ERα-dependent transcriptional activity [42]. Analysis of DNA binding sites indicated that ERβ can also bind DNA without ERα-interference in cells that express both receptors [67]. On the other hand, ERβ was found to interact with mutant p53 in triple-negative breast cancer cells indicating potential involvement of the receptor in the regulation of ERα-wild-type p53 transcriptional complex in luminal phenotypes [7]. Our findings demonstrate that ERβ interacts with wild-type p53 and attenuates the inhibitory effect of ERα on p53 function in luminal cells. The effect of ERβ on p53-ERα association may be explained by its direct association with either p53 or ERα and suggests that competition between ER subtypes for cooperation with p53 at the transcriptional level may occur in cells that express both receptors. Thus, the ratio of ERβ versus ERα and their affinity for p53 binding are crucial factors in determining p53 activity in estrogen responsive tissues. Our results point toward a role of ERβ as co-regulator that preserves p53 tumor suppressor activity. To achieve this, the receptor can differently act on wild-type and mutant p53 due to their formation of distinct transcriptional complexes. Only mutant p53 interacts with p63 and p73 and because of its impaired DNA binding activity, it often tethers to specific DNA sequences through other transcription factors. By binding to anti-metastatic p63 and preventing its normal transcriptional activity, mutant p53 promotes cell invasion [68, 69]. Our findings suggest that ERβ binds to both wild-type and mutant p53 [7]. In highly metastatic TNBC cells, the interaction of ERβ with mutant p53-p63 complexes attenuates the inhibitory effect of mutant p53 on p63 allowing p63 transcriptional activation that decreases invasion [7]. In luminal cells, ERβ acts on wild-type p53-ERα complex and increases the expression of the direct p53 target anti-proliferative and pro-apoptotic genes. In addition to TNBC cells, ERβ is highly likely to interact with mutant p53 in luminal tumor cells. This interaction may also depend on ERα and reverse gain-of-function activities of mutant p53 similarly with the repression of mutant p53 in ERβ-expressing TNBC cells [7]. Previous studies have shown that ERβ inhibits the growth both *in vitro* and *in vivo* of luminal T47D cells that express mutant p53 [70]. Consistent with these studies, our results show that ERβ increases the expression of the anti-proliferative p53 target genes suggesting that ERβ may restore the wild-type function of mutant p53. Our findings shed light onto the mechanism of p53 regulation in breast cancer. Delineating the effects of ER subtypes on p53 activity may advance methods of predicting therapy responses given that the ERβ/ERα ratio was previously proposed to function as a determinant of clinical outcome.

## MATERIALS AND METHODS

### Cell culture and reagents

ERα-positive and wild-type p53 breast cancer MCF-7 and ZR-75-1 cells lines and ERα-negative breast epithelial cell line MCF-10A were obtained from ATCC (Manassas, VA, USA). MCF-7 and ZR-75-1 cells were cultured in Dulbecco's Modified Eagle Medium (DMEM) supplemented with 10% fetal bovine serum (FBS). MCF-10A cells were cultured in DMEM/Nutrient Mixture F-12 media supplemented with 10% FBS, insulin and epidermal growth factor (EGF). The ERα-positive, estrogen-independent and tamoxifen-resistant MCF-7 cells (MCF-7-RR) were obtained from Dr. R. Clarke (Georgetown University) [71]. MCF-7-RR cells were maintained in phenol red-free Iscove's modified Eagle medium media (Invitrogen, Carlsbad, CA, USA) supplemented with 5% dextran-coated charcoal-stripped (DCC) FBS and 4-hydroxytamoxifen (Sigma-Aldrich, St Louis, MO, USA). The ERα-positive and p53 mutant T47D cell line was obtained from ATCC and cultured in Roswell Park Memorial Institute medium (RPMI1640) supplemented with 10% FBS. Stable cell lines were generated using pLenti6/V5 empty vector and pLenti6/V5-ERβ-Flag recombinant plasmid as previously described [72]. Empty pIRES vector and pIRES-ERβ plasmid were used for transient transfection. Previously validated siRNAs targeting ERβ (1# 5’-TTACGACATTAAGTAGTGTCGTCCC-3’ and 2# 5’-TATTGACCGCTACCTGGTGATTTCC-3’) were purchased from Invitrogen and doubly transfected to enhance ERβ downregulation [58]. An siRNA against luciferase was used as a control (Cat. No. 12935-146, Invitrogen).

### Ligand and drug treatments

To assess ER activity, breast cancer cells were maintained in 1% DCC-FBS media for 48 hours prior to treatment for 24 hours with 17β-estradiol (E2), diarylpropionitrile (DPN), fulvestrant (ICI182780 or ICI) or 4-hydroxytamoxifen (4-OHT). To induce genotoxic stress, cisplatin was freshly dissolved in DMSO and used at concentrations of 10 μM or 20 μM. Cells were incubated in the presence or absence of cisplatin and the ER subtype-specific ligands for 24 hours.

### RNA extraction and real-time reverse transcription (RT)-PCR

Cells were seeded in 6-well plates and total RNA was extracted using the Aurum Total RNA Mini Kit (Biorad). Copy DNA was generated from purified mRNA using the iScript cDNA Synthesis Kit (Biorad) and real-time PCR was conducted using the VeriQuest Fast SYBR Green qPCR Master Mix (Affymetrix). All primers used in real-time PCR are listed in Supplementary Table 2.

### Immunofluorescence analysis

Cells were maintained on coverslips for 48 hours in 1% DCC media followed by treatment for 24 hours with vehicle, cisplatin or cisplatin and DPN. Cells were then fixed with 3.7% paraformaldehyde (PFA) in PBS, permeabilized with 0.3% Triton X-100 in PBS, and blocked with 2% bovine serum albumin (BSA). Samples were incubated with anti-p53 (DO1-Santa Cruz) and anti-ERβ (14C8, Genetex) overnight at 4°C and probed with the secondary antibodies for 1 hour at room temperature. Coverslips were mounted with Vectashield medium containing DAPI for nucleus detection. Fluorescent images were acquired using an Olympus FV1200 inverted confocal microscope and the ERβ-p53 co-localization was analyzed using Pearson's correlation coefficient of 10 frames (123.993 μm X 123.993 μm) per sample with an Olympus FV10 Software.

### Co-immunoprecipitation and immunoblotting

All co-immunoprecipitation, pull-down and immunoblotting assays were performed as previously described [7]. Briefly, GST-tagged proteins were produced in bacteria (Rosetta) and solubilized by sonication combined to a freeze and thaw cycle. Glutathione sepharose beads were used for protein purification, followed by *in vitro*-translated protein immunoprecipitation. Proteins were immunoblotted after electrophoresis using specific antibodies. p53 was detected with DO1 antibody, ERβ with 14C8, the polyclonal 51-7700 (Invitrogen) or anti-FLAG (Cell signaling) antibodies. For the GST pull-down experiment, whole bacterial lysate was stained with SimplyBlue SafeStain (ThermoFisher) to detect protein expression.

### Survival assay

Cells were maintained in 1% DCC media for 48 hours in 96-well plates. Cells were exposed to specific treatments for 72 hours and survival was measured using a colorimetric CellTiter 96® AQueous One Solution Cell Proliferation MTS Assay as recommended by the manufacturer (Promega, US). The absorbance was measured using a plate reader at 490 nm.

### Analysis of clinical data

DNA sequencing datasets of chromatin immunoprecipitated ERβ and p53 (GSE42348 and GSE47041, respectively) were downloaded from Gene Expression Omnibus (GEO/NCBI). As previously described [73, 74], ERβ was expressed at similar level to endogenous ERα in MCF-7 cells (GSM1038224) or in ERα-knockdown MCF-7 cells (GSM1038225). Protein enrichment was analyzed using MACS (Model-based Analysis of ChIP-Seq) and common target genes were identified. Survival analysis was performed using Kaplan-Meier Plotter [53].

### Statistical analysis

Student's t test, ANOVA and Pearson correlation coefficient were used for statistical analysis. P-value <0.05 was considered significant.

## SUPPLEMENTARY MATERIALS FIGURES AND TABLES


