# Supplementary material for: ERβ alters the chemosensitivity of luminal breast cancer cells by regulating p53 function
Source: Oncotarget. 2018 Apr 27;9(32):22509–22. doi: 10.18632/oncotarget.25147 (PMC5976481; doi:10.18632/oncotarget.25147)
Supplement: Supplementary file 1 [file oncotarget-09-22509-s001.pdf]

ERβ alters the chemosensitivity of luminal breast cancer cells by regulating p53 function

SUPPLEMENTARY MATERIALS

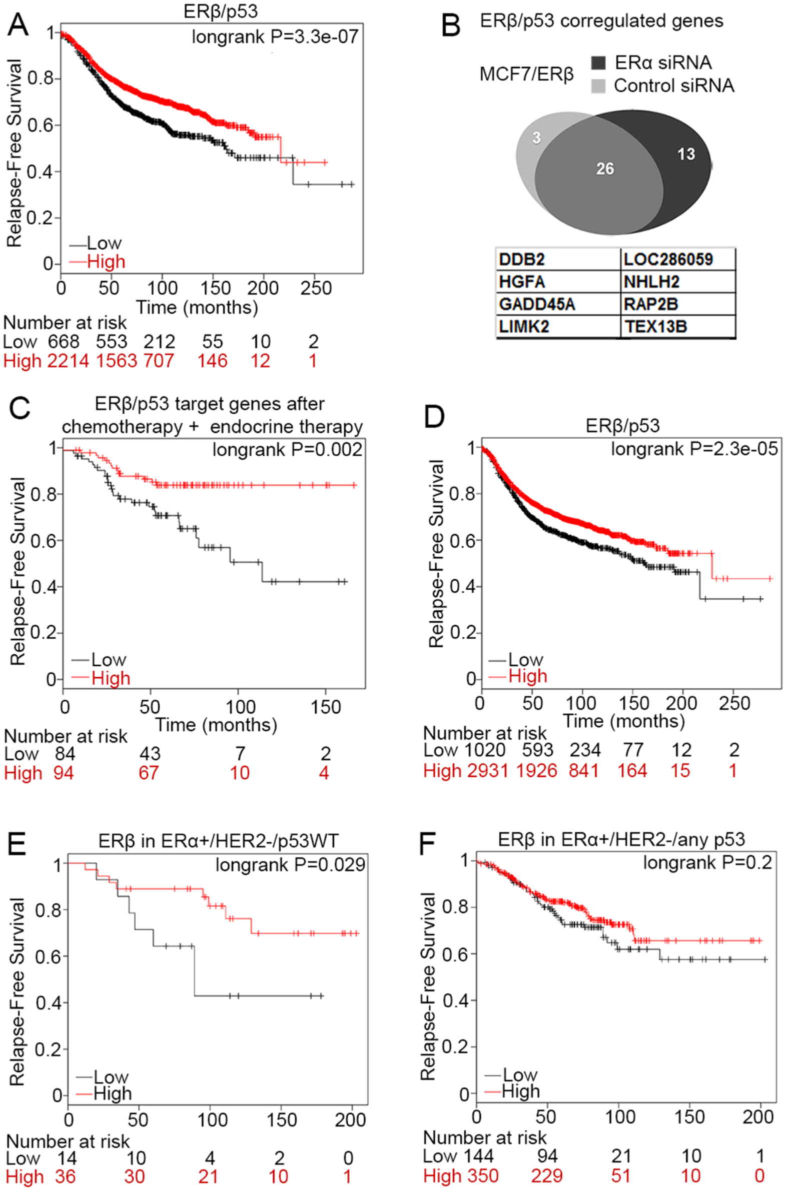

Supplementary Figure 1: ERβ and p53 signature correlates with better prognosis. (A) Correlation of dual ERβ and p53 mRNA expression with survival in ERα-positive breast cancer patients using KM plotter survival platform. **(B)** Venn Diagram from ChIP-seq analysis of ERβ and p53 common target genes (top). ERβ and p53 co-regulated genes with positive clinical correlation (bottom). Publically available data were obtained for GEO/NCBI database. **(C)** KM plot showing association of mRNA expression of ERβ/p53 common target genes (GADD45A, PUMA, p21, PAI-1) with survival in ERα-positive breast cancer patients following endocrine therapy and chemotherapy. **(D)** KM plot showing correlation of dual ERβ and p53 mRNA expression with survival in breast cancers that were treated with endocrine therapy. **(E, F)** KM plots showing association of ERβ mRNA expression with survival in ERα-positive/HER2-negative breast cancers that have either wild-type p53 (E) or any p53 (F) status.

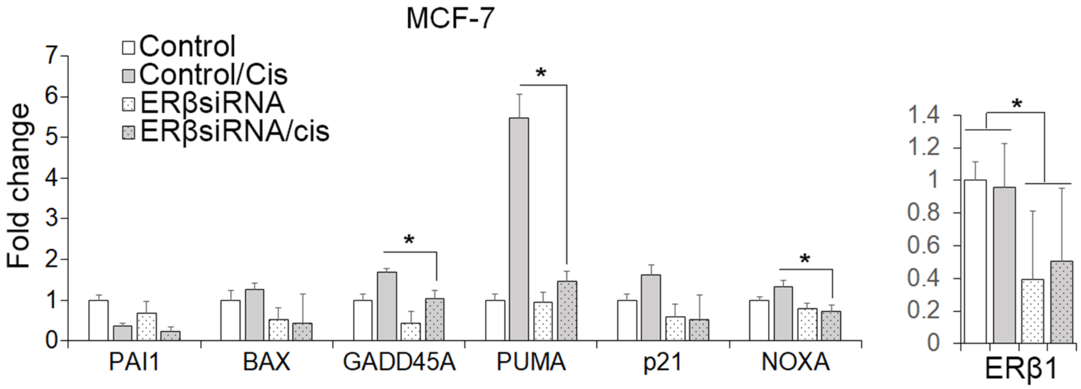

Supplementary Figure 2: Downregulation of ERβ alters p53-dependent transcription in response to chemotherapy. mRNA expression of p53 target genes in MCF-7 cells following transfection with control (Control) or siRNA#2 against ERβ and treatment with vehicle or 20 μM cisplatin for 24 hours. Values were normalized to the untreated control cells.

Supplementary Table 1: Genes bound to both ERβ and p53 in MCF-7 cells

ERβ-expressing MCF-7 cells		ERβ-expressing MCF-7 cells	
**Control**	**ERα siRNA**	**Control**	**ERα siRNA**
LOC101928973	LOC101928973	REV3L	**GADD45A**
C9orf69	C9orf69	NEURL1B	LOC286059
SPTSSA	DDB2	ACTG1	DAPL1
MDM2	ACTB	STC2	NEAT1
TRIAP1	SPTSSA	C17orf82	LOC101928855
FGD3	MDM2	SLC25A5-AS1	REV3L
ACTB	FGD3	PNPLA7	ACTG1
PPP4R3A	RRM2B	SULF1	LIMK2
9-Sept	HGFAC	KRT18	NEURL1B
PTGFRN	PPP4R3A		APPBP2
UNQ6494	FAM72C		STC2
PPM1D	9-Sept		C17orf82
MAD1L1	RAP2B		SLC25A5-AS1
TNFSF8	UNQ6494		TOB1
TP53INP1	MAD1L1		PNPLA7
ELF3	NHLH2		TEX13B
DDIT4	TNFSF8		SULF1
DAPL1	TP53INP1		HSPB1
NEAT1	ELF3		KRT18
LOC101928855	DDIT4		

Supplementary Table 2: Primers used in real-time PCR

Gene	FW	RV
PUMA	AACTGAAAAAGAAACGGAATGGAA	CTCCCTGGGGCCACAAATC
BAX	GCCCTTTTGCTTCAGGGTTTC	TGAGACACTCGCTCAGCTTC
PAI1	ACAACCCCACAGGAACAGTC	GATGAAGGCGTCTTTCCCCA
PML	TGGACGAGAACCTTGCTGAC	GCTGGCTAATCTTCTGGGTTTC
GADD45A	AAGGATGGATAAGGTGGGGGA	CACGTTATCGGGGTCGACGTT
p53	ACGCTTCCCTGGATTGGCAGC	GAGGGGGCTCGACGCTAGGA
RAP2B	AAAAGCCAAACGCATCCGAC	CTGGCCAATGCAAAGTGGTC
GADD45A	ACGATCACTGTCGGGGTGTA	CCACATCTCTGTCGTCGTCC
p21	CCAGCCTCTGGCATTAGAATT	CGGGATGAGGAGGCTTTAAAT
